# Plasma Total Homocysteine Levels in Diabetic Retinopathy

**DOI:** 10.1155/2014/758634

**Published:** 2014-08-14

**Authors:** Huseyin Kayadibi, Erdim Sertoglu, Metin Uyanik

**Affiliations:** ^1^Adana Military Hospital, Medical Biochemistry Laboratory, 01150 Adana, Turkey; ^2^Ankara Mevki Military Hospital, Anittepe Dispensary, Biochemistry Laboratory, 06580 Ankara, Turkey; ^3^Department of Medical Biochemistry, Gulhane School of Medicine, 06010 Ankara, Turkey

We read with great interest the recently published article by Malaguarnera et al. [1]. This study was aimed at evaluating the plasma total homocysteine (tHcy) levels in diabetic patients with and without retinopathy for the progression of diabetic retinopathy. In conclusion, higher plasma levels of tHcy have been found in diabetic patients with proliferative diabetic retinopathy compared to both nonproliferative diabetic retinopathy (DR) and diabetics without retinopathy. However, we would like to share our thoughts and contributions to the original study.

First, there was a significant difference between patient groups in terms of age, in the original study. However, there are age specific normal ranges for total homocysteine (tHcy) levels [2]. This is critical because comparison of patient groups with different age distributions may lead to a bias in tHcy results arising from different normal values by age. It should be better to select age-matched patient groups in such studies evaluating parameters with age specific normal values.

Second, specimens for tHcy analysis may be fresh or frozen plasma. Since red blood cells continue to produce and release homocysteine after blood sample has been obtained, plasma must be separated promptly [3]. Freshly drawn K2EDTA tubes must be kept in ice water, and plasma should be harvested within 30 min after drawing. Although this may affect the results directly, adding these details about sampling and storage procedure increases the quality of the original study.

Third, elevated tHcy levels cause inflammation by increasing arachidonic acid and the proinflammatory prostaglandin E2 [4]. However, tHcy levels in the blood are strongly influenced by diet. Dietary folic acid and vitamins B6 and B12 have the greatest effects [5]. However, none of these vitamins' levels were evaluated in the original study. Moreover, tHcy itself alone without other inflammatory markers (C-reactive protein, erythrocyte sedimentation rate, tumor necrosis factor-alpha, interleukin 6, etc.) may not accurately provide information about the presence of inflammation.

In conclusion, plasma total homocysteine (tHcy) levels are important in patients with diabetic retinopathy.
